# Diagnostic value of immunoglobulin G antibodies against *Candida* enolase and fructose-bisphosphate aldolase for candidemia

**DOI:** 10.1186/1471-2334-13-253

**Published:** 2013-05-31

**Authors:** Fang-qiu Li, Chun-fang Ma, Li-ning Shi, Jing-fen Lu, Ying Wang, Mei Huang, Qian-qian Kong

**Affiliations:** 1 Laboratory of Molecular Biology, Institute of Medical Laboratory Sciences, Jinling Hospital, School of Medicine Nanjing University, Nanjing 210002, P R China

**Keywords:** Candidemia, Serodiagnosis, *Candida* enolase, *Candida* fructose-bisphosphate aldolase, IgG antibody

## Abstract

**Background:**

The yeast *Candida* is one of the most frequent pathogens isolated from bloodstream infections and is associated with significant morbidity and mortality. Problems with clinical and microbiological diagnosis of invasive candidiasis (IC) have prompted the development of non-culture-based laboratory methods. Previous reports suggest that serological detection of antibodies might be useful for diagnosing systemic candidiasis.

**Methods:**

Diagnosis of IC using antibodies against recombinant *Candida albicans* enolase (Eno) and fructose-bisphosphate aldolase (Fba1) was evaluated. Using recombinant Eno and Fba1 as coating antigens, enzyme-linked immunosorbent assays (ELISAs) were used to analyze sera from patients with candidemia (n = 101), *Candida* colonization (n = 50), bacteremia (n = 84), invasive aspergillosis (n = 40); and from healthy controls (n = 200).

**Results:**

The results demonstrated that ELISA detection of anti-Eno and anti-Fba1 IgG distinguished IC from other pathogenic infections in patients and healthy individuals. The sensitivity, specificity, and positive and negative predictive values were 72.3%, 94.7%, 78.5% and 93% for anti-Eno, and 87.1%, 92.8%, 76.5% and 96.4% for anti-Fba1 antibodies, respectively. Combining these two tests improved sensitivity up to 90.1% and negative predictive value up to 97.1%, with specificity and positive predictive values of 90.6% and 72.2%. The tests were specific to the *Candida* genus and antibody titers were higher for candidemia patients than for controls. Positive antibody tests were obtained before blood culture results for 42.2% of patients for anti-Eno and 51.1% for anti-Fba1.

**Conclusion:**

These data suggest that tests that detect IgG antibodies against *Candida* enolase and fructose-bisphosphate aldolase, especially when used in combination, could be a powerful tool for diagnosing IC.

## Background

*Candida* species are among the pathogens most frequently isolated from bloodstream infections, and are associated with significant morbidity and mortality [1,2]. Infection by *Candida* species can involve any organ with severity ranging from mucosal or cutaneous infections to lethal invasive disease. In addition to hematological disease, prolonged hospitalization with treatment by broad spectrum antibiotics, intravascular catheters, intensive care unit hospitalization and gastrointestinal surgery put patients at substantial risk of invasive infections [3,4].

Since the signs and symptoms of invasive candidiasis (IC) are nonspecific, diagnosis remains a challenge. A positive blood culture is considered the gold standard for candidemia diagnosis. However, a relatively low sensitivity (less than 50% according to previous studies), especially in the early period of infection, dramatically hampers the value of blood culturing in clinical practice [5]. We observed that in some cases, a patient with a positive blood culture will test negative without anti-fungal treatment after removal of intravenous catheters. Thus, positive blood cultures can also be caused by fitted intravascular catheters rather than invasive infection (data not shown). The recovery of *Candida* from sputum is usually considered to indicate colonization, but should not be treated with antifungal therapy [6]. Other standard techniques for IC diagnosis including microscopic visualization of the infecting fungus and histopathology, usually lack specificity or sensitivity or require invasive procedures that cannot be performed because of the patient’s condition [5].

Recently investigations have focused on serological tests for diagnosing IC. Detection of *Candida* antigens and antibodies in serum samples are of practical clinical value [7-18]. Antibody assays are commercially available for (1 → 3)-β-D-glucan (BDG), a polysaccharide cell wall component of many fungi, and for Candida mannan antigen and antimannan. A meta-analysis [19] reported variable diagnostic performance for BGD assays with sensitivity 50%–85% and specificity 80%–99%. Individually, sensitivities were 58% for mannan antigen and 59% for antimannan antibody assays. When used together, assay sensitivity increased to 83% with no significant reduction in specificity [20]. Therefore, IC diagnosis is now recommended to be by serological tests for different antigens and antibodies used in combination.

The specific antibody response to *Candida* proteins that is usually induced in both immunocompromised and immunocompetent patients by invasive *Candida* infections is helpful in diagnosis [9,21]. However, antibody detection methods have limitations. First, methods traditionally used to detect antibodies are based on crude antigenic fungal extracts that usually show crossreactivity and low reproducibility. Second, immunocompromised patients can produce low or undetectable levels of antibodies, leading to false negative results. These problems could be solved, at least in part, by using suitable antigens and developing more sensitive antibody detection techniques [17,18]. Advances in molecular biology, genomics, proteomics, and bioinformatics are resulting in new strategies for more sensitive and specific diagnostic tests [22-24]. Using serological proteome analysis, 15 immunogenic proteins from lysates of *C. albicans* protoplasts were identified and differentially immunorecognized by serum IgG antibodies from IC patients compared to controls. This result provides candidate antigens that can be produced in large amounts in a prokaryotic host, making standardization easier and eliminating crossreactivity from posttranslational modifications [23]. In another study, enzyme-linked immunosorbent assays (ELISAs) measured serum antibody responses against recombinant *C. albicans* antigens in patients with IC and uninfected controls [18]. The results suggest that IgG response to a panel of *Candida* antigens might be an accurate and early marker of IC. Among the IgG antibodies, those against enolase (Eno) and fructose-bisphosphate aldolase (Fba1) showed high specificity and sensitivity in ELISA. Eno and Fba1 are well-studied, phase-specific proteins that are expressed on the cell wall of the germ tubes and hyphae of *C. albicans*[23]. Their location on the cell surface and loose association with the cell wall might facilitate the production of antibodies by *C. albicans-*infected hosts [24,25]. Eno and Fba1 are glycolytic and fermentation enzymes that catalyze opposing reactions during gluconeogenesis. They might be required for IC virulence and could serve as vaccine proteins [26-28]. Since the kinetics of antibody production vary among patients [17], detection of a combination of antibodies against different antigens in a single assay could optimize diagnosis.

In this study, we report the serodiagnosis of infection by invasive *Candida* species using ELISA to detect specific antibodies against recombinant Eno and Fba1. The specificities of each marker in patients with candidemia, *Candida* colonization, bacteremia, and invasive aspergillosis were confirmed; the sensitivities of detecting a single marker and a combination of two markers were also compared.

## Methods

### Patients and control subjects

All patients were admitted to Jingling hospital, Nanjing, China, from January 2009 to December 2011. Four non-overlapping patient groups were identified: (1) the Candidemia group (n = 101), (2) the Candida colonization group (n = 50), (3) the bacteremia group (n = 84), (4) the invasive aspergillosis (IA) group (n = 40). Below are definitions in this study. (1) Candidemia was defined as the isolation of *Candida* species from the bloodstream in at least one blood culture. (2) *Candida* colonization was defined as the presence of a positive *Candida* species culture from sputum only, none of these patients had a positive blood culture for Candida species but positive for other organisms. (3) the bacteremia was microbiologically defined by positive culture of blood and (4) IA was defined according to the EORTC-MSG criteria [29]. The sera (n = 200) of healthy control subjects were from the volunteers who did not have any clinical or microbiological evidence of infection. The study protocol was approved by the Ethics Committee of Jingling Hospital and informed consent was obtained from all patients included in the study. All data, including age, primary condition, and clinical stage, outcome of hospital stay were obtained from the clinical records.

### Collection of sera

All of the serum samples drawn for clinical laboratory tests from each candidemia patients were collected for our study. The first serum was obtained one week before the first positive culture presented. Subsequently, serum samples were obtained twice a week until the patient being discharged or died. Serum samples from 50 patients with *Candida* colonization and 84 patients with bacteremia were collected on the date of positive culture. Sera of IA patients were from our previous research [30,31]. All sera were stored at −80°C.

### Identification of organisms

The blood was inoculated into a BacT-ALERT aerobic vial (Becton Dickinson). All positive cultures were manually sampled and inoculated on CHROMagar Candida medium (Hardy Laboratories, Santa Maria, Calif.) to ensure viability and purity. An aliquot was Gram-stained for preliminary identification of the microorganism. Identification of all species was confirmed with the Vitek-2 system (bioMerieux, France).

### Generation of recombinant Eno and Fba1

Primers were designed to clone and express full-length protein. 5′-TACCATATGTCTTACGCCACTAAAATCC-3′ and 5′-ACTGGATCCTTACAATTGAGAAGCCTTTGG-3′ for enolase, and (5′-GCACATATGGCTCCTCCAGCAGTTTTAAG −3′) and (5′-GCAGGATCCTTACAATTGTCCTTTGGTGTG −3′) for fructose-bisphosphate aldolase. PCR products were cloned into the pET-28a (+) expression vector (Novagen, Germany). All inserts were confirmed by DNA sequencing. The plasmids were transformed into *Escherichia coli* BL21 (DE3) cells (Novagen). Expression of recombinant antigens was induced by isopropyl-β-D- thiogalactopyranoside (IPTG). His_6_-tagged recombinant proteins were confirmed by 15% SDS-PAGE, which showed a single band of the expected size. Recombinant antigens were purified from cell-free supernatants by chromatography on Ni^2+^ -nitrilotriacetic acid-agarose in accordance to manufacturer’s instructions (Novagen). Fractions containing purified recombinant proteins were pooled, dialyzed against PBS and stored at −70°C.

### ELISA

Preliminary checkerboard titration experiments were performed to determine the optimal concentration of antigen by comparing known positive and negative human sera. Microtiter plates (Costar, Cambridge, Mass, USA) were coated with purified recombinant proteins (1 μg/ml). Antigen was diluted in 0.05 M sodium carbonate buffer (pH 9.6) and 100 μl was added to each well before incubating at 4°C overnight. After coating, wells were washed once with PBS-T (PBS containing 0.5% Tween 20) and blocked with PBS-T containing 5% bovine serum albumin (BSA) for 1 h at 37°C. Sera were diluted 1:500 in PBS-T and assayed in triplicate (100 μl/well). After 1 h at 37°C, wells were washed three times with PBS-T. Then, 100 μl of a 1:10,000 dilution of horseradish peroxidase (HRP)-conjugated goat antihuman IgG (Signalwayantitibody, USA, L3012) in PBS-T was added to each well and plates were incubated at 37°C for 1 h. Plates were washed three times with PBS-T followed by 100 μl of substrate solution (3,3′,5,5′-tetramethylbenzidine, TMB) per well. Plates were incubated at room temperature for 20 min in darkness. Reactions were stopped by 50 μl per well of 0.5 M sulphuric acid and absorbance was measured at 450 nm using an automated ELISA plate reader (Microplate reader, Bio-Rad Model 680). The mean absorbance for each serum was calculated.

The block assay was used to confirm the specificity of the ELISA. The sera that were intensively positive by ELISA were diluted and incubated with 2.5 μg/ml recombinant antigens at 37°C for 1 h before ELISA. In order to check the His-tag cross-reactivity, His_6_-Eno and His_6_-Fba1 were replaced with His_6_-TR (a recombinant protein of *A. fumigatus* with low homology to most other fungi) [30] as coating antigen in ELISA.

### Statistical analysis

For continuous variables, differences were analyzed by the Mann–Whitney *U* test. For categorical variables, a chi-square test was employed. A *p* values less than 0.05 was considered statistically significant. Sensitivity of diagnostic techniques was calculated from proven cases. Specificity was calculated from control groups. Statistical analysis was performed using SPSS for Windows version 16.0 (SPSS Inc.).

## Results

### Study population and sera collection

The study population was classified by age, gender, underlying disease, risk factors, and outcome of hospital stay (Table 1). *Candida* isolates were classified to the species level (Table 2). The number of sera per patient was varying, from 2 to 25. So, 1015 serum samples were obtained from 101 patients with candidemia. The sera from 84 patients with bacteremia were obtained, the bacteria infected including 47 Gram-positive coccal species: 36 *Staphylococcus*, 4 *Streptococcus*, 4 *Enterococcus*, 3 other Gram-positive cocci; 26 Gram-negative bacilli species: 11 *Escherichia coli*, 5 *Klebsiella pneumonia*, 3 *Acinetobacter baumannii*, 3 *Serratia marcescens*, 4 *Pseudomonas aeruginosa*; and 11 other gram negative bacilli.

**Table 1:** Base-line characteristics of the 475 subjects and results of serum testing

	**Number [anti-Eno (%) positive; anti-Fba1(%) positive]**				
		**Control group**			
**Characteristic**	**Patients with candidemia**	**Patients with candida colonization**	**Patients with bacteremia**	**Patients with IA**^**a**^	**Healthy subjects**
	**n = 101**	**n = 50**	**n = 84**	**n = 40**	**n = 200**
Demographic factors					
Sex					
Male	63 (73.0, 82.5)^f^	41 (7.3; 7.3)	52 (3.8,19.2)	33 (6.1; 0)	117 (2.6; 5.1)
Female	38 (71.1, 94.7)	9 (22.2; 22.2)	32 (6.3; 9.4)	7 (14.3; 14.3)	83 (1.2; 2.4)
Age (years, mean ± SD)	52.3 ± 19.6	76.12 ± 14.89	51.9 ± 14.2	57.6 ± 15.8	59.1 ± 12.4
≤65 years	71 (69.0, 87.3)^g^	9 (22.2; 44.4)	48 (6.3; 14.6)	28 (3.6; 0)	108 (1.9; 4.6)
>65 years	30 (80.0, 86.7)	41 (7.3; 2.4)	36 (2.8; 16.7)	12 (16.7; 8.3)	92 (2.2; 3.3)
Primary condition					
Hematological malignancy	10 (60.0; 80.0)	3 (33.3; 33.3)	6 (33.3; 33.3)	1 (0; 0)	0
Leukemia	5 (40.0; 80.0)	1 (0; 0)	3 (33.3; 33.3)		
Lymphoma	3 (100.0; 66.7)	1 (100.0; 100.0)	2 (50.0; 50.0)		
Myelodysplasia	1 (0; 100.0)	1 (0; 0)	1 (0; 0)		
Multiple myeloma	1 (100.0; 100.0)	0 (0; 0)	0 (0; 0)	1 (0; 0)	
Solid tumor	11 (45.5; 81.8)	7 (14.3; 28.6)	10 (0; 30.0)	0	0
Bronchopulmonary neoplasm	2 (50.0; 50.0)	1 (0; 0)	4 (0; 50.0)		
Pancreas/colon adenocarcinoma	7 (71.4; 85.7)	6 (16.7; 33.3)	5 (0; 20)		
Bladder neoplasm	2 (50.0; 100.0)	0 (0; 0)	1 (0; 0)		
Nonmalignant diseases	80 (77.5; 88.8)	40 (7.5; 5.0)	68 (2.9; 11.8)	39 (7.7; 2.6)	0
Respiratory dysfunction^b^	5 (80.0; 60.0)	18 (11.1; 5.6)	3 (33.3; 33.3)	21 (4.8; 4.8)	
Gastrointestinal pathology^c^	65 (75.4; 92.3)	4 (25.0; 25.0)	60 (1.7; 11.7)	2 (100.0; 0)	
Others^d^	10 (90.0; 80.0)	18 (0; 0)	5 (0; 0)	16 (0; 0)	
Risk factors					
Iatrogenic predisposing factors					
Broad spectrum antibiotics	68 (76.5; 86.8)^h^	44 (4.5; 2.3)	64 (4.7; 9.4)	20 (5.0; 5.0)	0
Glucocorticoids therapy	57 (87.7; 91.2)	40 (2.5; 2.5)	46 (2.2; 2.2)	0 (0; 0)	0
Central venous catheters	36 (72.2; 88.9)	28 (0; 0)	27 (0; 0)	9 (0; 0)	0
Parenteral nutrition	56 (82.1; 92.9)	15 (0; 0)	49 (0; 0)	3 (66.7; 0)	0
Other risk factors					
Intensive care unit stay	26 (80.8; 92.3)^i^	12 (8.3; 16.7)	18 (5.6; 11.1)	12 (16.7; 0)	0
Neutropenia^e^	11 (36.4; 45.5)	2 (0; 50.0)	3 (0; 33.3)	0 (0; 0)	0
Acute renal failure	2 (50.0; 50.0)	2 (50.0; 0)	1 (100.0; 0)	0 (0; 0)	0
Outcome of hospital stay					
Death	36 (63.9; 88.9)^j^	8 (37.5; 50.0)	10 (20.0; 50.0)	12 (8.3; 8.3)1,1	n^k^
Discharge	65 (76.9; 86.2)	42 (4.8; 2.4)	74 (2.7; 10.8)	28 (7.1; 0)	n^k^

^a^ Patients with proven and probable IA.

^b^Includes the following diseases: pneumonia, chronic obstructive pulmonary disease, and acute respiratory distress syndrome.

^c^ Includes the following diseases: cholecystitis, angiocholitis, pancreatitis, peritonitis, and hepatitis.

^d^ Includes the following diseases: multiple trauma, acute renal insufficiency, and diabetes mellitus.

^e^ Neutropenia was defined as an absolute neutrophil count below 500 cells/mm^3^.

^f,g,h,I,j^ No significant difference was found in patients with candidemia associated with age, sex, predisposing factors for IC and clinical outcomes (*P* > 0.05).

^k^ Not applicable.

**Table 2:** **IgG antibodies and microbiological surveillance of patients with candidemia and *Candida *colonization**

	**Anti-Eno**				**Anti-Fba1**			
**Microorganism**	**Candidemia**		***Candida *colonization**		**Candidemia**		***Candida *colonization**	
	**n**	**No. (%) positive**	**n**	**No. (%) positive**	**n**	**No. (%) positive**	**n**	**No. (%) positive**
*C. albicans*	25	21 (84.0)	37	1 (2.7)	25	23 (92.0)	37	2 (5.4)
*C. tropicalis*	20	9 (45.0)	7	1 (14.3)	20	16 (80.0)	7	2 (28.6)
*C. parapsilosis*	21	18 (85.7)	1	0 (0)	21	19 (90.5)	1	0 (0)
*C. glabrata*	10	7 (70.0)	3	2 (66.7)	10	8 (80.0)	3	1 (33.3)
*C.guilliermondii*	5	3 (60.0)	—	—	5	5 (100.0)	—	—
*Candida spp.*	20	15 (75.0)	2	1 (50.0)	20	17 (85.0)	2	0 (0)
Total	101	73 (72.3)^a^	50	5 (10.0)	101	88 (87.1)^b^	50	5 (10.0)

_*X*_^2^_a_ =51.94, *P* < 0.01; _*X*_^2^_b_ =84.1, *P* < 0.01, when compared with the control (patients with *candida* colonization).

### Characterization of recombinant Eno and Fba1

**Figure 1:**
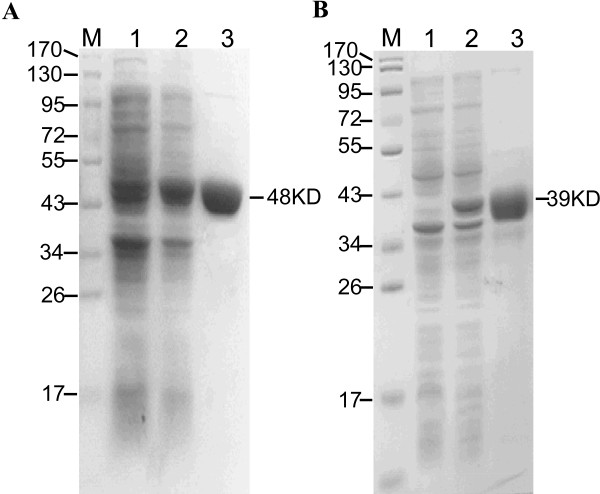
**SDS-PAGE of recombinant enolase (Eno) and fructose-bisphosphate aldolase (Fba1). A**. Lanes: 1, pET28a-Eno in *E. coli* BL21; 2, pET28a-Eno in *E. coli* BL21, induced by IPTG; 3, purified recombinant Eno. **B**. Lanes: 1, pET28a-Fba1 in *E. coli* BL21; 2, pET28a-Fba1 in *E. coli* BL21, induced by IPTG; 3, purified recombinant Fba1. Molecular markers (in kDa) of standard proteins are to the left.

Recombinant antigens were expressed as His6-tagged proteins in *E. coli* cells and appeared as single bands of expected sizes by SDS-PAGE, with molecular mass of 48 kDa for Eno and 39 kDa for Fba1 (Figure 1).

### Analytical validation of ELISA assays

Serum samples from 101 patients with candidemia and 374 serum samples from control groups were tested. The cut-off value for serum anti-Eno and anti-Fba1 was set by receiver operating characteristic curves with *A* = 0.370 for anti-Eno and *A* = 0.610 for anti-Fba1. Patient sera were regarded as positive when ELISA results were above these cut-off points at a serum dilution of 1:500. Sera with positive test results were diluted two-fold until the test results became negative. Titers were defined as the highest serum dilution with a positive result. The specificity of the ELISA was confirmed by the block assay. The absorbance was reduced by 93.0% for anti-Eno and 85.3% for anti-Fba1 after intensively positive sera incubated with antigens. When His_6_-Eno and His_6_-Fba1 were replaced with His_6_-TR, no substantial His_6_-tag cross-reactivity was detected.

### Detection of antibodies in patients

**Figure 2:**
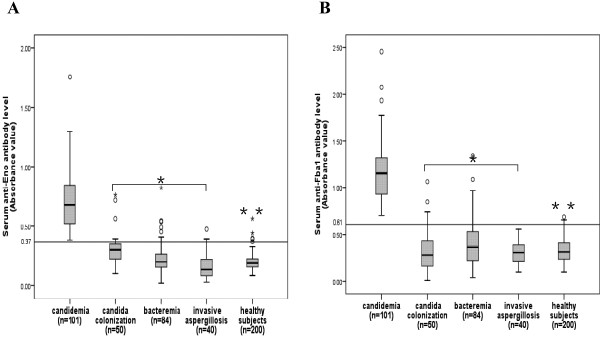
**Antibody levels in study patients (A: anti-Eno and B: anti-Fba1).** Patients with candidemia, *Candida* colonization, bacteremia, invasive aspergillosis and healthy controls were evaluated for the presence of anti-Eno and anti-Fba1 antibodies. The levels of anti-Eno and anti-Fba1 in patients with candidemia were higher than control groups. Boxes indicate interquartile ranges (25–75th percentiles). Horizontal bars in bold indicate the median value in each group. Whiskers extend to 1.5 times the interquartile range. ^*^P < 0.001, for the comparison with the patients with candidemia; ^**^P < 0.001, for the comparison with the patients with candidemia.

A total of 1015 serum samples from 101 patients with candidemia were tested. The median anti-Eno antibody absorbance was significantly higher for sera from candidemia patient (0.682; interquartile range, 0.522–0.849) than for sera from non-IC patients (0.282; interquartile range, 0.175–0.368; *P* < 0.001) or healthy individuals (0.188; interquartile range, 0.156–0.227; *P* < 0.001). Likewise, candidemia patients had a greater prevalence of seropositive anti-Fba1 antibody (1.156; interquartile range, 0.930-1.322) than non-IC patients (0.317; interquartile range, 0.189-0.488; *P* < 0.001) or healthy individuals (0.316; interquartile range, 0.235-0.410; *P* < 0.001), as shown in Figure 2. Serological and microbiological surveillance of patients with candidemia and *Candida* colonization are in Table 2.

**Figure 3:**
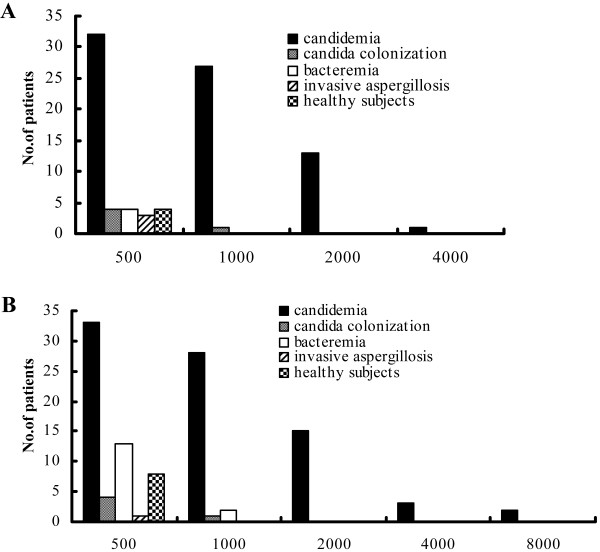
**Anti-Eno and anti-Fba1 antibody titers in patients with candidemia and control groups. A**, anti-Eno titers; **B**, anti-Fba1 titers. In 73 anti-Eno positive patients, 32 at a titer of 1:500, 41 at titers ≥1:1000. In 88 anti-Fba1 positive patients, 81 were tested titers, 33 at a titer of 1:500, 48 at titer ≥1:1000. In 15 anti-Eno positive control individuals, only one at a titer of 1:1000. In 26 anti-Fba1 positive control individuals, 3 at a titer of 1:1000. None control individuals at titer >1:1000.

The anti-Eno and anti-Fba1 antibody titers in candidemia patients were significantly higher than in control groups (Figure 2 and Figure 3). Although anti-Eno and anti-Fba1 antibodies were detected in some individuals in the control groups at a titer of 1:500, greatly reduced in number at titers 1: 1000 (only one patients with *Candida* colonization and two pateint with bacteremia), these samples were all negative at titers >1: 1000. No significant differences in these levels were found among control groups. Patients infected with C. albicans and control groups were more likely to have antibody against Fba1, in *C. albicans*-infected patients, 84% (21/25) were positive for anti-Eno and 92% (23/25) were positive for anti-Fba1, while in non-*C. albicans* infected patients, 68.4% (52/76) were positive for anti-Eno and 85.5% (65/76) were positive for anti-Fba1, but no significant differences were found between groups (84% vs 68.4%, *X*^2^ = 2.28, *p* > 0.05; 92% vs 85.5%, *X*^2^ = 0.703, *p* > 0.05). For candidemia, neither the seroprevalence nor the titers of the identified antibodies were significantly associated with age, sex, and predisposing factors for IC, causative *Candida* species, or clinical outcomes (Table 1). The combined detection of anti-Eno and anti-Fba1 (sera gave single or both assay positive result were regarded as positive, Table 3) gave a sensitivity of 90.1%, specificity of 90.6%, positive predictive value of 72.2%, and negative predictive value of 97.1%. The availability of serial consecutive serum samples from candidemia patients allowed us to study the kinetics of the antibody response to Eno and Fba1 by ELISA. Anti-Eno and anti-Fba1 antibodies were detected in 50% of non-neutropenia patients before the diagnosis of candidemia made blood culture. However, the seroprevalence of antibodies was reduced in neutropenia patients (Table 4).

**Table 3:** Diagnostic value of anti-Eno and anti-Fba1 on the test cohort

	**Anti-Eno**	**Anti-Fba1**	**Combination of anti-Eno and anti-Fba1**
True negative	354	347	339
False positive	20	27	35
True positive	73	88	91
False negative	28	13	10
Sensitivity (%)	72.3	87.1	90.1
Specificity (%)	94.7	92.8	90.6
Negative predictive value (%)	93.0	96.4	97.1
Positive predictive value (%)	78.5	76.5	72.2

The number of patients is presented in the upper part of the table. Sensitivity, specificity, and positive and negative predictive values are estimated in percentage calculated from patient responses. In the combined detection of anti-Eno and anti-Fba1, sera with single or both assay positive results were regarded as positive.

**Table 4:** **Positive rate and timing of anti-Eno and anti-Fba1 antibodies in non-neutropenic and neutropenic patients with candidemia [*n *(%)]**

**Population (No. of subjects)**	**Anti-Eno**			**Anti-Fba1**		
	Ab^* ^**(+) prior to BC(+)**	BC^**^(+) prior to Ab(+)	**Total**	**Ab(+) prior to BC(+)**	**BC(+) prior to Ab(+)**	**Total**
Non-neutropenia (90)	38 (42.2)	31 (34.4)	69 (76.7)	46 (51.1)	37 (41.1)	83 (92.2)
Neutropenia^#^ (11)	3 (27.3)	1 (9.1)	4 (36.4)^a^	4 (36.4)	1 (9.1)	5 (45.5)^b^
Total (101)	41 (40.6)	32 (31.7)	73 (72.3)	50 (49.5)	38 (37.6)	88 (87.1)

^*^Ab: antibody, ^**^ BC: blood culture.

^#^Neutropenia was defined as an absolute neutrophil count below 500 cells/mm^3^.

^a^*P* = 0.005, for the comparison with the Non-neutropenia group.

^b^*P* < 0.001, for the comparison with the Non-neutropenia group.

## Discussion

We report the development of two ELISAs for serodiagnosis of invasive *Candida* infections. The assays detect specific IgG antibodies against recombinant Eno and Fba1. We evaluated the diagnostic value of the assays by comparing patients and diverse control participants. We measured antibodies against Eno and Fba1 in patients with candidemia, *Candida* colonization, bacteremia, and invasive aspergillosis; and in healthy controls. The results revealed that both Eno and Fba1 were specific for the *Candida* genus, and that recombinant antigens from *C. albicans* reacted with sera from candidemia patients infected with *C. albicans* and other *Candida* species including *C. tropicalis*, *C. parapsilosis*, *C. glabrata*, *C. guilliermondii*. Both the seroprevalence and serum levels of anti-Eno and anti-Fba1 in candidemia patients were significantly higher than in control groups (Table 1 and Figure 3). ELISA specificity was confirmed by block assay.

Several studies on the diagnostic utility of detecting antibodies against Eno have had promising results [9,10,18,32]. Láın et al. [9] described the performance of a diagnostic ELISA to detect antibodies against recombinant enolase in 98 patients (47 immunocompromised and 51 immunocompetent). The results were similar in both patient groups, with sensitivities of 78.9% in immunocompromised and 82.6% in immunocompetent patients, and specificities of 89.3% and 78.6%. These results confirm the utility of detecting recombinant enolase antibodies for diagnosis of invasive candidiasis, even in immunocompromised patients. Our study detected anti-Eno and anti-Fba1 antibodies in 90 non-neutropenic and 11 neutropenic patients with candidemia. Of these, 76.7% (69/90) of the non-neutropenic and 36.4% (4/11) of the neutropenic patients were seropositive. Our results suggested that the diagnostic value of anti-Eno and anti-Fba1 antibodies was limited in neutropenic patients (Table 4). Clancy et al. [18] reported antibody responses against 15 recombinant antigens from 12 proteins in 60 patients with systemic candidiasis and 24 uninfected controls. IgM and IgG titers against the recombinant antigens were consistently detected by ELISA. For all 15 antigens, IgG responses were superior to IgM responses for discriminating patients with systemic candidiasis from controls. IgG titers were detectable for anti-Eno in 98.3% (59/60) of participants with system candidiasis and for anti-Fba1 in 93.3% (56/60); IgM titers were detectable for anti-Eno in only 46.7% (28/60) of these patients and for anti-Fba1 in only 36.7% (22/60). Using discriminating analysis that included IgG responses against the 15 antigens, a mathematical prediction model was developed to identify patients with systemic candidiasis. The model had an an error rate of 3.7%, a sensitivity of 96.6%, and a specificity of 95.6%. Our study included more patients and controls than previous studies. For anti-Eno, sensitivity was 72.3%, specificity was 94.7%, positive predictive value was 78.5% and negative predictive values was 93%; for anti-Fba1, these values were 87.1%, 92.8%, 96.4% and 76.5%, respectively (Table 3). While the performance of each assay alone was unremarkable, when the two tests were combined, the sensitivity improved to 90.1% and and negative predictive value improved to 97.1%. When developing disease screening strategies, maximizing the negative predictive value is important for reliably identifying patients who are not likely to develop the disease. Therefore, the combination of the anti-Eno and anti-Fba1 assays was a favorable screening tool for IC.

In this study, collection of serial serum samples was initiated one week before positive blood culturing, and ended at discharge or death of the patient. In about 50% patients, we obtained a positive detection of antibody earlier than the positive blood culture. The explanation for this result is that in our hospital, anti-fungal agents are prescribed to patients with clinical suspicion of IC before a positive blood culture is received. Anti-fungal therapy might produce a false-negative blood culture result but should have no effect on an antibody test. Anti-Fba1 usually appeared slightly earlier than anti-Eno, but the mechanism of this difference is unknown. We also found that serum IgG responses against selected two *Candida* antigens discriminated candidemia from exclusively *Candida* positive sputum cultures. These results suggested that, patients with non-*Candida* infections, only patients with positive sputum cultures might have similar levels of anti-Eno and anti-Fba1.

## Conclusion

In conclusion, our findings suggested that anti-Eno and anti-Fba1 could be useful screening tools and early markers of IC.

## Competing interests

The authors declare that they have no competing interests.

## Authors’ contributions

FQL conceived, coordinated and designed the study, and contributed to the acquisition, analysis and interpretation of data and drafted the manuscript. CFM, LNS and JFL performed the experiment and involved in drafting the article. YW, MH and QQK participated in sample collection and data acquisition. All the authors have read and approved the final manuscript.

## Pre-publication history

The pre-publication history for this paper can be accessed here:

http://www.biomedcentral.com/1471-2334/13/253/prepub

## References

[B1] Tortorano AM Dho G Prigitano A Breda G Grancini A Emmi V Cavanna C Marino G Morero S Ossi C Delvecchio G Passera M Cusumano V David A Bonaccorso G Corona A Favaro M Vismara C Garau MG Falchi S Tejada MR Invasive fungal infections in the intensive care unit: a multicentre, prospective, observational study in Italy (2006–2008) Mycoses 2012 55 1 73 79 10.1111/j.1439-0507.2011.02044.x 21668521

[B2] Kourkoumpetis T Manolakaki D Velmahos G Chang Y Alam HB De Moya MM Sailhamer EA Mylonakis E Candida infection and colonization among non-trauma emergency surgery patients Virulence 2010 1 5 359 366 10.4161/viru.1.5.12795 21178471

[B3] Zirkel J Klinker H Kuhn A Abele-Horn M Tappe D Turnwald D Einsele H Heinz WJ Epidemiology of Candida blood stream infections in patients with hematological malignancies or solid tumors Med Mycol 2012 50 1 50 55 10.3109/13693786.2011.587211 21696259

[B4] Mikulska M Bassetti M Ratto S Viscoli C Invasive candidiasis in non-hematological patients Mediterr J Hematol Infect Dis 2011 3 1 e2011007 21625311 10.4084/MJHID.2011.007 PMC3103237

[B5] Yera H Sendid B Francois N Camus D Poulain D Contribution of serological tests and blood culture to the early diagnosis of systemic candidiasis Eur J Clin Microbiol Infect Dis 2001 20 12 864 870 10.1007/s100960100629 11837637

[B6] Hacimustafaoglu M Celebi S Candida infections in non-neutropenic children after the neonatal period Expert Rev Anti Infect Ther 2011 9 10 923 940 10.1586/eri.11.104 21973304

[B7] Morrison CJ Hurst SF Reiss E Competitive binding inhibition enzyme-linked immunosorbent assay that uses the secreted aspartyl proteinase of Candida albicans as an antigenic marker for diagnosis of disseminated candidiasis Clin Diagn Lab Immunol 2003 10 5 835 848 12965914 10.1128/CDLI.10.5.835-848.2003 PMC193877

[B8] Takazono T Izumikawa K Nagayoshi Y Tanaka A Mihara T Kosai K Saijo T Imamura Y Miyazaki T Seki M Kakeya H Yamamoto Y Yanagihara K Kamihira S Kohno S Evaluation of the Cica Fungi Test Candida, a novel serum Candida mannan antigen kit, and its comparison with Cand-Tec in candidemia patients Jpn J Infect Dis 2011 64 2 116 120 21519124

[B9] Laín A Moragues MD Ruiz JC Mendoza J Camacho A Del Palacio A Pontón J Evaluation of a novel enzyme-linked immunosorbent assay to detect immunoglobulin G antibody to enolase for serodiagnosis of invasive candidiasis Clin Vaccine Immunol 2007 14 3 318 319 10.1128/CVI.00396-06 17229884 PMC1828855

[B10] Pitarch A Jiménez A Nombela C Gil C Serological proteome analysis to identify systemic candidiasis patients in the intensive care unit: Analytical, diagnostic and prognostic validation of anti-Candida enolase antibodies on quantitative clinical platforms Proteomics Clin 2008 2 4 596 618 10.1002/prca.200780039 21136858

[B11] Yang Q Su QP Wang GY Wen DZ Zhang YH Bao HZ Wang L Production of hybrid phage displaying secreted aspartyl proteinase epitope of Candida albicans and its application for the diagnosis of disseminated candidiasis Mycoses 2007 50 3 165 171 10.1111/j.1439-0507.2006.01349.x 17472610

[B12] Zaragoza R Pemán J Quindós G Iruretagoyena JR Cuétara MS Ramírez P Gómez MD Camarena JJ Viudes A Pontón J Clinical significance of the detection of Candida albicans germ tube-specific antibodies in critically ill patients Clin Microbiol Infect 2009 15 6 592 595 10.1111/j.1469-0691.2009.02794.x 19438621

[B13] Lu Y Chen YQ Guo YL Qin SM Wu C Wang K Diagnosis of invasive fungal disease using serum (1 → 3)-β-D-glucan: a bivariate meta-analysis Intern Med 2011 50 22 2783 2791 10.2169/internalmedicine.50.6175 22082890

[B14] Laín A Elguezabal N Brena S García-Ruiz JC Del Palacio A Moragues MD Pontón J Diagnosis of invasive candidiasis by enzyme-linked immunosorbent assay using the N-terminal fragment of Candida albicans hyphal wall protein 1 BMC Microbiol 2007 7 35 10.1186/1471-2180-7-35 17448251 PMC1868733

[B15] Philip A Odabasi Z Matiuzzi G Paetznick VL Tan SW Warmington J Rex JH Ostrosky-Zeichner L Syscan3, a kit for detection of anti-*Candida* antibodies for diagnosis of invasive candidiasis J Clin Microbiol 2005 43 9 4834 4835 10.1128/JCM.43.9.4834-4835.2005 16145152 PMC1234065

[B16] Lau A Chen S Sleiman S Sorrell T Current status and future perspectives on molecular and serological methods in diagnostic mycology Future Microbiol 2009 4 9 1185 1222 10.2217/fmb.09.70 19895220

[B17] Laín A Elguezabal N Amutio E Fernández De Larrinoa I Moragues MD Pontón J Use of recombinant antigens for the diagnosis of invasive candidiasis Clin Dev Immunol 2008 2008 721950 18382617 10.1155/2008/721950 PMC2276615

[B18] Clancy CJ Nguyen ML Cheng S Huang H Fan G Jaber RA Wingard JR Cline C Nguyen MH Immunoglobulin G responses to a panel of candida albicans antigens as accurate and early markers for the presence of systemic candidiasis J Clin Microbiol 2008 46 5 1647 1654 10.1128/JCM.02018-07 18322056 PMC2395065

[B19] Morrissey CO Advancing the field: evidence for new management strategies in invasive fungal infections Curr Fungal Infect Rep 2013 7 1 51 58 10.1007/s12281-012-0128-4 23420637 PMC3568482

[B20] Mikulska M Calandra T Sanguinetti M Poulain D Viscoli C The use of mannan antigen and anti-mannan antibodies in the diagnosis of invasive candidiasis: recommendations from the third European conference on infections in leukemia Crit Care 2010 14 6 R222 10.1186/cc9365 21143834 PMC3219989

[B21] Klingspor L Stintzing G Tollemar J Deep Candida infection in child liver transplant recipients: serological diagnosis and incidence Acta Paediatr 1995 84 4 424 428 10.1111/j.1651-2227.1995.tb13664.x 7795354

[B22] Pitarch A Abian J Carrascal M Sánchez M Nombela C Gil C Proteomics-based identification of novel Candida albicans antigens for diagnosis of systemic candidiasis in patients with underlying hematological malignancies Proteomics 2004 4 10 3084 3106 10.1002/pmic.200400903 15378761

[B23] Pitarch A Jimenez A Nombela C Gil C Decoding serological response to Candida cell wall immunome into novel diagnostic, prognostic, and therapeutic candidates for systemic candidiasis by proteomic and bioinformatic analyses Mol Cell Proteomics 2006 5 1 79 96 16195222 10.1074/mcp.M500243-MCP200

[B24] Pitarch A Sánchez M Nombela C Gil C Sequential fractionation and two-dimensional gel analysis unravels the complexity of the dimorphic fungus Candida albicans cell wall proteome Mol Cell Proteomics 2002 1 12 967 982 10.1074/mcp.M200062-MCP200 12543933

[B25] Montagnoli C Sandini S Bacci A Romani L La Valle R Immunogenicity and protective effect of recombinant enolase of Candida albicans in a murine model of systemic candidiasis Med Mycol 2004 42 4 319 324 10.1080/13693780310001644653 15473356

[B26] Rodaki A Young T Brown AJ Effects of depleting the essential central metabolic enzyme fructose-1,6-bisphosphate aldolase on the growth and viability of *Candida albicans*: implications for antifungal drug target discovery Eukaryot Cell 2006 5 8 1371 1377 10.1128/EC.00115-06 16896220 PMC1539134

[B27] Cutler JE Corti M Lambert P Ferris M Xin H Horizontal transmission of Candida albicans and evidence of a vaccine response in mice colonized with the fungus PLoS One 2011 6 7 e22030 10.1371/journal.pone.0022030 21818288 PMC3139608

[B28] Xin H Cutler JE Vaccine and monoclonal antibody that enhance mouse resistance to candidiasis Clin Vaccine Immunol 2011 18 10 1656 1667 10.1128/CVI.05215-11 21832099 PMC3187024

[B29] De Pauw B Walsh TJ Donnelly JP Stevens DA Edwards JE Calandra T Pappas PG Maertens J Lortholary O Kauffman CA Revised definitions of invasive fungal disease from the European organization for research and treatment of cancer/invasive fungal infections cooperative group and the national institute of allergy and infectious diseases mycoses study group (EORTC/MSG) consensus group Clin Infect Dis 2008 46 12 1813 1821 10.1086/588660 18462102 PMC2671227

[B30] Shi LN Li FQ Huang M Lu JF Kong XX Wang SQ Shao HF Immunoproteomics based identification of thioredoxin Reductase GliT and novel Aspergillus fumigatus antigens for serologic diagnosis of invasive Aspergillosis BMC Microbiol 2012 12 11 10.1186/1471-2180-12-11 22251604 PMC3398318

[B31] Shi LN Li FQ Lu JF Kong XX Wang SQ Huang M Shao HF Shao SH Antibody specific to thioredoxin reductase as a new biomarker for serodiagnosis of invasive aspergillosis in non-neutropenic patients Clin Chim Acta 2012 413 9–10 938 943 22366166 10.1016/j.cca.2012.02.011

[B32] van Deventer AJ van Vliet HJ Hop WC Goessens WH Diagnostic value of anti-Candida enolase antibodies J Clin Microbiol 1994 32 1 17 23 8126174 10.1128/jcm.32.1.17-23.1994 PMC262962

